# Vitamin D and Depression: A Systematic Review and Meta-Analysis Comparing Studies with and without Biological Flaws

**DOI:** 10.3390/nu6041501

**Published:** 2014-04-11

**Authors:** Simon Spedding

**Affiliations:** Nutritional Physiology Research Centre, University of South Australia, City East Campus, North Tce, Adelaide, SA 5000, Australia; E-Mail: spedding@adam.com.au; Tel.: +61-439-687-866; Fax: +61-882-900-498

**Keywords:** Vitamin D supplementation, depression, biological plausibility, meta-analysis, systematic review, 25OHD

## Abstract

Efficacy of Vitamin D supplements in depression is controversial, awaiting further literature analysis. Biological flaws in primary studies is a possible reason meta-analyses of Vitamin D have failed to demonstrate efficacy. This systematic review and meta-analysis of Vitamin D and depression compared studies with and without biological flaws. The systematic review followed the Preferred Reporting Items for Systematic Reviews and Meta-Analyses (PRISMA) guidelines. The literature search was undertaken through four databases for randomized controlled trials (RCTs). Studies were critically appraised for methodological quality and biological flaws, in relation to the hypothesis and study design. Meta-analyses were performed for studies according to the presence of biological flaws. The 15 RCTs identified provide a more comprehensive evidence-base than previous systematic reviews; methodological quality of studies was generally good and methodology was diverse. A meta-analysis of all studies without flaws demonstrated a statistically significant improvement in depression with Vitamin D supplements (+0.78 CI +0.24, +1.27). Studies with biological flaws were mainly inconclusive, with the meta-analysis demonstrating a statistically significant worsening in depression by taking Vitamin D supplements (−1.1 CI −0.7, −1.5). Vitamin D supplementation (≥800 I.U. daily) was somewhat favorable in the management of depression in studies that demonstrate a change in vitamin levels, and the effect size was comparable to that of anti-depressant medication.

## 1. Introduction

Depression affects 350 million people worldwide, is the leading cause of disability and the fourth-leading cause of the global disease burden [1]. However, the effectiveness of conventional treatments for depression is questioned: meta-analyses of drug treatments demonstrate minimal difference from placebo, comparisons of real and sham electroconvulsive therapy show little difference after a month, and the evidence for the use of specific cognitive interventions is weak [2]. Therefore we examined the evidence for other approaches to the management of depression. 

The association between depressive disorders and Vitamin D deficiency from a lack of sun exposure is well established and was first noted two thousand years ago [3], therefore we considered the evidence for the effectiveness of Vitamin D supplementation.

Vitamin D is a unique secosteroid hormone formed mainly by photosynthesis, so an indoor lifestyle and sun-avoidance leads to deficiency (25OHD <50 nmol/L) [4]. Vitamin D deficiency is now a global public health problem affecting a billion people worldwide [5]. Even in sunny Australia, deficiency affects one third of the population [6], with much higher rates observed in migrant populations [7,8]. There has been an increase in the prevalence of Vitamin D deficiency [9] and a ten-fold increase in spending on supplements in the US over the last decade [10].

Knowledge of Vitamin D has grown exponentially [11] and 95% of our current knowledge was published in the last 15 years [12]. This demonstrates new mechanisms and diseases associated with deficiency including cancer, cardiovascular disease, diabetes, and premature mortality [4]. Whilst Vitamin D was believed to follow Funk’s model of vitamins, having a single mechanism and function limited to calcium and bone metabolism [13], the mechanisms of action of Vitamin D are now recognized to be endocrine, paracrine and autocrine via Vitamin D receptors (VDRs) [14] affecting most physiological systems, including the brain [15]. The enzymes necessary for the hydroxylation of 25hydroxyvitamin D (25OHD) to the active form 1,25dihydroxyvitamin D are present in the hypothalamus, cerebellum, and substantia nigra [16]. Vitamin D modulates the hypothalamic-pituitary-adrenal axis, regulating adrenalin, noradrenaline and dopamine production through VDRs in the adrenal cortex [17]; and protects against the depletion of dopamine and serotonin centrally [18]. Therefore, biological plausibility for the action of Vitamin D in depression has been established.

Epidemiological evidence shows that Vitamin D deficiency is associated with an 8%–14% increase in depression [19,20,21,22] and a 50% increase in suicide [23]; however, causality and efficacy of supplementation remain controversial [10,24] awaiting confirmation by systematic review and meta-analysis.

Four systematic reviews of Vitamin D efficacy in depression, but no meta-analysis, have been published [25,26,27,28]. These reviews provide conflicting results due to the limited number of studies found and the inclusion of inappropriate studies. Based on six RCTs deemed relevant, the Institute of Medicine (IOM) [25] concluded there was “inconclusive evidence of an effect” although four of these RCTs showed a beneficial effect of Vitamin D supplementation in depression. The inclusion of the other two studies [29,30] described by the IOM as “RCTs of Vitamin D” was inappropriate as; one used calcium and not Vitamin D as the intervention, and the other was not an RCT in the opinion of the study authors as the intervention decreased 25OHD levels. Similarly, consistent conclusions could not be drawn from the other systematic reviews [26,27,28], as these found so few of the primary studies.

These reviews mirror the inconsistent results found across Vitamin D research as demonstrated by the twenty four conflicting meta-analyses for falls, fractures, and all-cause mortality [31]. The reason Vitamin D meta-analyses fail to produce useful results is thought to be biological flaws in primary studies. These flaws lead to null results [32] as the intervention does not change the Vitamin D status however these flaws may be overlooked when evaluating the research for Vitamin D and other nutrients [33,34].

The concept of “biological flaws” arises from the work of Heaney and others [33,34], and refers to limitations in the design of primary studies which preclude them from testing the research hypothesis. The hypothesis being addressed in this review is that rectifying Vitamin D deficiency decreases depressive symptoms. However some trials have limitations in their study design that prevent this evaluation. This hypothesis can only be tested if participants are Vitamin D deficient at baseline and then receive a large enough dose of Vitamin D supplements to achieve Vitamin D sufficiency during the trial. Vitamin D deficiency cannot be demonstrated if the level of 25OHD is sufficient or higher or not tested at baseline. An ineffective dose of Vitamin D is one that would not be expected to increase the level of 25OHD from deficient to sufficient.

Trials with these biological flaws may demonstrate the limitations of the study design rather than the effectiveness of Vitamin D supplements for changing health outcomes. The parallel in pharmaceutical research to these nutrient studies with biological flaws would be trialling a drug known to be ineffective or on patients already taking a full dose of the drug. Thus biological flaws are a critical element that differentiates nutrient research from pharmaceutical research.

This review was designed to estimate the effect of Vitamin D supplementation in depression and examine the influence of biological flaws in primary studies on the meta-analyses.

## 2. Methods

This review followed the PRISMA (Preferred Reporting Items for Systematic Reviews and Meta-Analyses) guidelines, systematically identifying and appraising peer-reviewed RCTs reporting on the effect of Vitamin D supplementation for individuals with symptoms of depression with the objectives of investigating:

- the primary evidence for Vitamin D supplementation and depression from RCTs;
- the types of subjects, the dose of Vitamin D supplementation, the control interventions and the measures of outcome used;
- methodological quality of the studies;
- biological flaws in the study design, and
- estimates of the size of the effect.

### 2.1. Search Approach

A systematic search for relevant RCTs was performed evaluating oral Vitamin D supplementation that included data on depression using four library databases of PsychINFO, MedLine, PubMed and Cochrane online library. Search approaches for the different databases can be obtained from the researchers. All databases were searched from inception to October 2012, with eligible papers limited to English language and human subjects.

### 2.2. Independence

Two independent researchers investigated the library databases to reduce errors/bias in accessing evidence. The reference lists of four systematic reviews [25,26,27,28] were hand-searched to identify other RCTs.

### 2.3. Eligible Studies

RCTs were included where the intervention was Vitamin D supplementation and excluded where trials were not RCTs or used surrogate interventions. Studies were not excluded on their methodological quality as the entire evidence base was required to address the aims of this research.

### 2.4. Decision-Making

Relevant publications were identified from title, abstract and study descriptors by one researcher; the decision to include was independently validated by a second and disagreements were referred to third for an independent ruling.

### 2.5. Critical Appraisal

Methodological quality of articles was critically appraised with PEDro [35]. Trials were rated with a checklist, the PEDro scale. This considers two aspects of trial quality; internal validity of the trial and whether the trial contains sufficient statistical information to make it interpretable. It does not rate external validity or the effect size.

### 2.6. Data Extraction

Data was extracted for participants, 25OHD levels, study timeframes, interventions, outcome measures, measures of effect, methodological quality scores, and biological flaws.

### 2.7. Biological Flaws

Biological flaws in primary studies were identified. These studies included:

- inappropriate interventions (interventions that did not include Vitamin D), or
- interventions producing the opposite effect of that intended (interventions that included Vitamin D, but reduced the 25OHD level in the intervention group), or
- ineffective interventions that did not improving Vitamin D status (did not significantly change the 25OHD level), or
- where the baseline 25OHD level was not measured in the majority of participants, or
- where the baseline 25OHD level indicated sufficiency (not deficiency) at baseline.

Studies were grouped according to the presence of biological flaws, and compared by date of publication, methodological quality, outcome measure, and study outcome.

### 2.8. Meta-Analysis

Meta-analyses were performed using MedCalc where data was available on diagnosis, dose, outcome measure, and biological flaws. Estimates of the size of effect using the standardised mean difference (SMD) were compared according to the presence of biological flaws in primary studies.

For meta-analysis of studies with a continuous measure, MedCalc uses the “Hedges g” statistic as a formulation for the SMD under the fixed effects model. The SMD is the difference between the two means divided by the pooled standard deviation, with a correction for small sample bias. Next the heterogeneity statistic is incorporated to calculate the summary SMD under the random effects model. The total SMD with 95% CI is given both for the Fixed effects model and the Random effects model.

The SMD has no units or dimensions, however using Cohen's rule of thumb for interpretation of the SMD statistic: a value of 0.2 indicates a small effect, a value of 0.5 indicates a medium effect, and a value of 0.8 or larger indicates a large effect.

## 3. Results

### 3.1. Systematic Review

From all databases 465 relevant articles were identified with 390 articles remaining after removal of duplicates. After applying inclusion criteria, 375 were removed and 15 articles remained. These included 15 RCTs [30,36,37,38,39,40,41,42,43,44,45,46,47,48,49], nine new RCTs and six identified by previous reviews. Seven of the 15 were published in 2011 and 2012 (Table 1).

There was wide variation in study methodology. The study populations were diverse (Table 1). Smaller studies were performed in patients with specific disorders (depression, seasonal affective disorder, obesity, post-menstrual tension and hospitalized patients) [30,37,38,39,41,42,43,44,47,48,49,44,47], and studies in University students [45,46].

**Table 1:** Study populations, sample sizes (numbers entering intervention and control groups respectively) and methodological quality score (PEDro Scale).

Author	Year	Reference Citation #	Population	Sample Size	Quality Score
Arvold *et al.*	2009	[36]	Individuals with Vit D deficiency (10–25 ng/mL) seen for medical care at a primary healthcare clinic	100 (I 50, C 50)	10
Belcaro *et al.*	2010	[42]	Menopausal women with signs of depression and mood disorder	65 (I 33, C 32)	8
Bertone-Johnson *et al.*	2012	[38]	PostmenopausalWomen with depressive symptoms	36,282 (I 18176, C 18106)	11
Dean *et al.*	2011	[45]	Young healthy adults (University students)	128 (I 63, C 65)	11
Dumville *et al.*	2006	[43]	Older women with seasonal affective disorder	2117 (I 912,C 1205)	11
Gloth *et al.*	1999	[44]	Adults with Season Affective Disorder	15 (I 8,C 7)	6.5
Harris & Dawson-Hughes	1993	[30]	Women with seasonal affective disorder	250 (I 125, C 125)	5
Jorde *et al.*	2008	[37]	Overweight and obese adults	441 (IH 150, ILl 142, C 149)	8
Khajehei *et al.*	2009	[46]	University female students with premenstrual syndrome	180 (IOes 60, I 60, C 60)	9
Khoraminya *et al.*	2013	[49]	Adults with major depressive disorder based on DSM-IV criteria, without psychosis	40 (I 20, C 20)	10
Landsdowne & Provost	1998	[39]	Adults with seasonal affective disorder	44 (I 22, C 22)	8
Sanders *et al.*	2011	[47]	Community dwelling older women with seasonal mood disorders	2012 (I 1001, C 1011)	11
Veith *et al.*	2004	[40]	Adults with serum 25(OH)D <61 nmol/L in summer, expected to develop 25(OH)D concentrations <40 nmol/L by winter	64 ( I 32, C 32)	10
Yalamanchilli & Gallagher	2012	[48]	Older post-menopausal women with depression	488 (Ioes+Calcitrol 122, Ioes 122, Calcitrol 123, placebo 123 )	11
Zhang *et al.*	2011	[41]	Hospitalized patients	32 (I 17, C 15)	9

C = control group and I = intervention group. Where there are two intervention groups; IH is used to indicate where a high dose and IL for where a low dose of Vitamin D supplements were given. Where one intervention group took a hormone, this was designated IOes.

Baseline 25OHD levels were not reported in six papers [36,37,38,39,40,41] but were performed in eight studies [42,43,44,45,46,47,48,49] (Table 2). For one study [30], Vitamin D data was sought from an earlier paper [50] showing 25OHD levels were not measured at baseline. However 25OHD levels were measured twice during the study. This demonstrated that the 25OHD levels decreased 5% in the intervention group during this part of the study due to the decreased availability of sunlight with the change in season, overwhelming the effect of the low dose of Vitamin D supplements provided.

**Figure 1:**
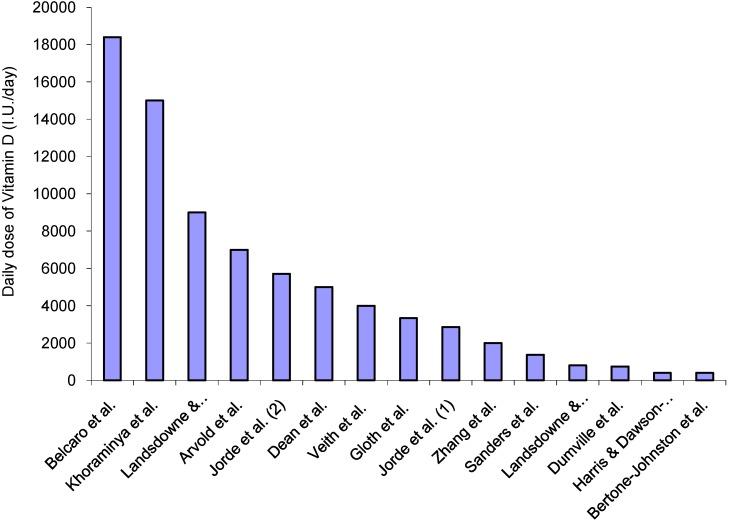
Daily dose of Vitamin D per study. This shows the range of equivalent daily doses. (These were calculated after estimating the actual dose rather than using the dose shown in their published papers).

Daily doses varied from 400 I.U. to 18,400 I.U. across the 15 trials (Figure 1). Three studies [30,38,43] used doses lower that 800 I.U./day. In the Women’s Health Initiative [38], the Vitamin D dose would be inadequate to change vitamin levels; the actual dose ingested was ≈200 I.U., as the stipulated dose was 400 I.U. but compliance was 46%. The doses shown in two papers were misprints; reported as 200 mg Vitamin D [42] and 0.25 g of calcitriol [48], equating to millions of international units. However, attempts to clarify this with authors and editors were unsuccessful. The intervention in another study [47] was high dose Vitamin D (500,000 I.U.) probably inducing side effects; a 15% increase in falls and 26% increase in fractures*.*

Low doses of 400 I.U. in Harris & Dawson-Hughes [30] and Bertone-Johnson *et al.* [38]; High doses were over 15,000 I.U. per day in Belcaro *et al.* [42] and Khoraminya *et al.* [49]; Jorde *et al.* [37] and Landsdowne & Provost [39] both tested three groups; two differing dosages and one placebo.

Validated outcome measures of depression (Table 2) included Beck Depression Index in three studies [37,45,49] the Profile of Mood States in two studies [30,41] and the mental component score of the SF12 in two studies [43,47]. Questionnaires about pre-menstrual syndrome [46], fibromyalgia [36], and menopause [42] included depression as a domain. One early study used an unvalidated questionnaire [39]. There was no significant differences at baseline measures and methodological quality of studies was generally high (9 out of 11) (Table 1).

**Table 2:** Key depression outcome measures, within and between group findings.

Author	Year	Outcome Measures	Follow-up Time Period	Within Group Findings	Between Group Findings	
Arvold *et al*.	2009	Fibromyalgia Impact Questionnaire	8 weeks	FIQ score Mean pre-post difference total (95%CI) intervention −3.71 (−7.5 to 0.1) (*p* < 0.03), control 1.91 (−2.9 to 6.7) (*p* > 0.05)	*p* < 0.05 favoring intervention	
Belcaro *et al*.	2010	Menopause Symptoms Questionnaire	8 weeks	Total average symptom score reduced by 48% for intervention group (*p* < 0.05), control group increased by 10% (*p* > 0.05).	*p* < 0.05 favoring intervention	
Bertone-Johnson *et al*.	2012	Burnam Depression Scale	At 2 weeks, then twice yearly for 2 years	Mean overall change (SD) 0.004 (0.143) intervention, −0.002 (0.113) (control)	*p* > 0.05	
Dean *et al*.	2011	Beck Depression Index	6 weeks	Baseline: follow up mean (95%CI): Intervention 7.24 (5.58–8.90); 6.40 (4.73–8.07) (*p* > 0.05); control 5.72 (4.09–7.36); 5.38 (3.74–7.02) (*p* > 0.05)	*p* > 0.05	
Dumville *et al*.	2006	SF12 mental component	6 months	Mean difference (95%CI) between intervention and control at baseline −0.6 (−1.5 to 0.3) (*p* > 0.05); at follow up 1.8 (−0.8 to 1.2) (*p* > 0.05)	Mean adjusted (age- and baseline score) between group difference (95%CI) −0.49 (−1.34 to 0.81) *p* > 0.05	
Gloth *et al*.	1999	SAD-8	1 month	Significant improvement in SAD-8 scores for intervention group, not control (explanatory data not provided)	Significant association between improvement in Vit D levels and SAD-8 scores in overall cohort (r^2^ = 0.26)	
Harris & Dawson-Hughes	1993	Profile of Mood States	3 monthly for 12 months	No difference in pre-post scores for any domain of PoMS for either intervention or control (*p* > 0.05)	No difference between intervention or control change over time in any domain (*p* > 0.05)	
Jorde *et al*.	2008	Beck Depression Index (total score)	12 months	Baseline: DD group 4.5 (0.0–24.0); DP group 5.0 (0.0–28.0); PP group 4.0 (0.0–24.0). Follow-up: DD group 3.0 (0.0–23.0) (*p* < 0.05); DP group 4.0 (0.0–26.0) (*p* < 0.05); PP group 3.8 (0.0–18.0)	DD and DP groups change was similar (*p* > 0.05) but significantly greater from PP (*p* < 0.05)	
Khajehei *et al*.	2009	PMS symptom rating form which captured psychological and physical symptoms including depression	Pre-mens for 2 cycles	Mean % total symptoms *Pre:* Dydrogesteron group 52.1%, Calcium plus Vitamin D group 50.7%, Placebo 53.7%. *Post* (respectively): 47.9%, 46.1%, 53.7%Both active treatment groups had significant decreases	The dydrogesterone and calcium plus Vitamin D treatments were significantly more effective than placebo in lessening the severity of PMS symptoms (*p* < 0.05)	
Khora-minya *et al*.	2013	24-item Hamilton Depression Rating Scale (HDRS) (1°), 21-item Beck Depression Inventory (BDI) (2°)	Every 2 weeks for 8 weeks	BDI Intervention Wk0 32.45 ± 7.35; Wk2 27.73 ± 7.50; Wk4 20.44 ± 6.56; Wk6 16.73 ± 8.11; Wk8 13.2 ± 8.64 (*p* < 0.05) Control. Wk0 31.65 ± 7.33; Wk2 29.17 ± 6.78; Wk4 25.18 ± 6.93; Wk6 21.00 ± 6.81; Wk8 17.95 ± 6.31 (*p* < 0.05)	*p* < 0.05 for both outcomes, favoring intervention	
Lands-downe & Provost	1998	PANAS	5 days	Sig within-group improvements for both active interventions (*p* < 0.05)	Sig improvements for both active interventions cf control for positive and negative affects (*p* < 0.05)	
Sanders *et al*.	2011	General Health Questionnaire SF12 (PCS, MCS), WHO Wellbeing Index	3–5 years	Intervention: no intervention SF12 PCS effect size (95%CI) 0.27 (−2.40 to 2.94) 0.23 (−0.88 to 1.34)	Treatment effects SF12 effect size (95%CI) PCS 0.22 (−70.75 to 1.19); MCS 70.14 (−71.00 to 0.72)	
Veith *et al*.	2004	Self-developed Wellbeing Scale	2–6 months	Pre-post mean (SD): *600 I.U.* 2.2 (2.0); 2.3 (2.3) (*p* > 0.05) *4000 I.U.* 2.0 (2.3); 1.1 (1.8) (*p* < 0.05)	Significant improvement in wellbeing, favoring higher Vit D dose	
Yalamanchilli & Gallagher	2012	Geriatric Depression Scale	1. HT alone 2. calcitriol alone 3. HT & calcitrol 4. placebo	*% with depression* (pre/post) 13.8%; 8.9%; 9.7%; 7.3%; 8.2%; 6.6% 13.8%; 8.9% All groups *p > 0.05*	No effect on depression in any treatment group compared with placebo (*p* > 0.05)	
Zhang *et al*.	2011	Profile of Mood States questionnaire	Average 8 days	Vit D group pre-post 23.1 ± 27.2; 22.4 ± 22.4 *p > 0.05* Vit C group pre-post 28.6 ± 21.8; 18.8 ± 19.4) *p < 0.05*	*p* < 0.05 favouring Vit D	

### 3.2. Biological Flaws

Biological flaws were found in eight of the 15 studies (Table 3). These flaws limit the ability of these studies to demonstrate a change in vitamin status in the intervention group. The most common flaw, occurring in five studies, was not measuring 25OHD. Two studies [30,38] utilized doses below the minimum effective dose of 600-800 I.U. [51] and one study [45] had such high baseline 25OHD levels that supplements could not improve the Vitamin D status of participants. One intervention was associated with a decrease in 25OHD level [30], and another caused falls and fractures minimising the potential to see any health benefits [47]. Biological flaws were more prevalent (70%) in recent studies (since 2010) than in earlier studies (50%), and in larger studies than in smaller studies (Table 3).

**Table 3:** Comparison of studies by presence of biological flaws to the study findings and methodological quality.

Study	Biological Flaws NOT Present	Biological Flaw(s) Present	Type of Flaw		Quality Score (Max 11)	Date of Publication	
			*25OHD not Assessed*	*Dose not Appropriate*			
Belcaro *et al.*		X	X		8		2010
Bertone-Johnson *et al.*		X	X	X (L)	11		2012
Dumville *et al.*		X	X		11	2006	
Harris & Dawson-Hughes		X	X	X (L)	5	1993	
Dean *et al.*		X	X	X (H)	11		2011
Khajehei *et al.*		X		X (I)	9	2009	
Sanders *et al.*		X		X (SE)	11		2011
Yalamanchilli & Gallagher		X		X (I)	11		2012
**Total-8 Studies with Biological Flaws**	**0**	**8**	**5**	**6**		**3**	**5**
Arvold *et al.*	**X**				***10***	2009	
Gloth *et al.*	**X**				6.5	1999	
Jorde *et al.*	**X**				8	2008	
Khoraminya *et al.*	**X**				*10*		2013
Landsdowne & Provost	**X**				8	1998	
Veith *et al.*	**X**				10	2004	
Zhang *et al.*	**X**				9		2011
**Total—7 studies without flaws**	7	**0**	**0**	**0**		**5**	**2**

↑ = significant improvement favouring Vitamin D; Dose incorrect (I), low (L), high (H) or produces side effects (SE).

Of the seven studies without flaws, six [36,37,39,40,44,49] showed improvement in depression with supplementation, whereas six of the nine flawed studies [30,38,42,45,46,47,48] had a null result (Table 3). The positive results in two flawed studies maybe due to the unknown contents [46] or the effects of the herbs [42] used in these studies.

### 3.3. Meta-Analysis

#### 3.3.1. Meta-Analysis of Studies without Biological Flaws (Right Panel of Figure 2)

Two studies (Jorde *et al.* [37] and Khoraminya *et al.* [49]) were included as they used the same outcome measure; the Beck Depression Inventory.

**Figure 2:**
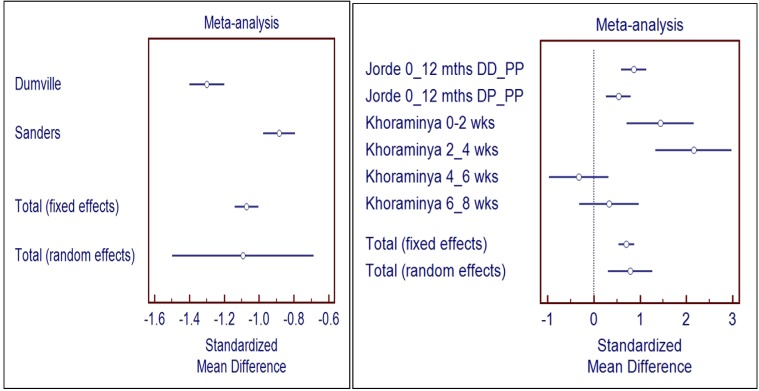
The figures show the meta-analysis of studies from the systematic review.

The standardized mean difference for these studies without flaws is shown in the Right Panel of Figure 2. It shows a statistically significant positive effect of Vitamin D in depression of 0.78 (CI 0.24, 1.27). The random effects model was used due to the diverse populations studied.

**Left Panel**—Two studies with biological flaws were combined, Dumville *et al.* [43] and Sanders *et al.* [47];

**Right Panel**—Two studies without biological flaws were combined, Jorde *et al.* [37] and Khoraminya *et al.* [49], showing two intervention groups for Jorde *et al.* [37] (high and low dose Vitamin D) and the data from the Khoraminya *et al.* [49] at 2, 4, 6, and 8 weeks.

The Jorde *et al.* [37] trial (*n* = 387) had three study groups; two interventions with different doses of Vitamin D and a control. The Khoraminya *et al.* [49] trial (*n* = 40) compared Vitamin D plus fluoxetine to fluoxetine alone. The studies had similar baseline level of 25OHD (Jorde *et al.* [37] 55 nmol/L) (Khoraminya *et al.* [49] 57 nmol/L), and the doses of Vitamin D over 800 nmol/L in both studies. The participants in both studies were patients; Khoraminya *et al.* [49] depressed patients and Jorde *et al.* [37] obese patients. Depression and obesity overlap, as there is a reciprocal relationship between obesity and depression indicated by the 50% increase in one condition when the other is present [52].

#### 3.3.2. Meta-Analysis of Studies with Biological Flaws (Left Panel of Figure 2)

Options for meta-analysis were examined and performed combining the Dumville *et al.* [43] and Sanders *et al.* [47] studies, due to the diverse outcome variables used in other studies. There was a statistically significant negative effect of Vitamin D administration evident from the forest plot in the standardized mean differences as shown in the Left Panel of Figure 2. The effect size was −1.1 (CI −0.7, −1.5) (random effects). These studies were of high methodological quality, had similar subjects (community dwelling women aged >70 years) and baseline 25OHD, and used the same outcome measure. The studies differed in the dosing schedule, daily and annually.

## 4. Discussion

This is the most comprehensive systematic review of randomized controlled trials investigating the effectiveness of Vitamin D in the management of depression. Fifteen RCTs were found, whilst previous reviews captured few of the available RCTs. Although the methodological quality was good, biological flaws were common and more prevalent in recent studies.

For the meta-analysis of studies without biological flaws, the size of the effect was statistically significant being +0.78 (CI 0.24, 1.27). As the measure of effect size was the standardized mean difference (SMD), this was 0.78, using Cohen’s Rule-of-Thumb, a SMD of 0.8 is considered to indicate a large effect.

As less than half the study population were deficient the effect of the intervention was diluted such that if all subjects had been deficient the size of the effect would have been higher, perhaps double, 1.5 points on the BDI scale. This is similar to the size of effect seen in a large RCT of antidepressant medication, which was 0.8 point on the BDI scale for the blinded parts of the study and 1.7 points overall [53]. A review of antidepressant efficacy published in the NEJM [54] shows that the effect size of antidepressant medication was increased by selective publication of trials and altering the effect size. However the overall mean weighted effect size value for antidepressants was only 0.15 (CI 0.08, 0.22) for unpublished studies and 0.37 (CI 0.33, 0.41) for published studies. Thus, the effect size of Vitamin D demonstrated in our meta-analysis may be comparable with that of anti-depressant medication. For the meta-analysis of studies with biological flaws, the size of the effect was statistically significant and negative being −1.1 (CI −0.7, −1.5), indicating that Vitamin D supplementation in flawed studies may lead to deterioration in depression.

The main finding is that all studies without flaws and the meta-analysis of studies without biological flaws support the efficacy of Vitamin D supplementation for depression, as compared with the negative results of meta-analysis for studies with biological flaws. The Womens Health Initiative [38] (WHI), with more participants that all the other studies combined, had the highest methodological quality and the most biological flaws leading to non-significant outcomes for both bone strength and mood. Due to its sheer size, the WHI has dominated previous meta-analysis leading to null results.

The main limitation of this review was the diversity of study methodology precluding more extensive meta-analyses, and leaving only two studies in each meta-analysis. The variability in outcome measures and reporting suggest agreement should be sought within the research community to underpin standard conduct and reporting of future studies to support meta-analysis.

## 5. Conclusion

Traditional evidence, biological plausibility and epidemiological studies indicate Vitamin D has therapeutic effects in depression. There are no previous meta-analyses of Vitamin D and depression as the evidence was deemed to be insubstantial [25]. This may be due to previous systematic reviews identifying few of the available studies and including RCTs with inappropriate methodology and biological flaws.

Meta-analysis of studies without biological flaws demonstrates that improving Vitamin D levels improves depression, whereas the meta-analysis of flawed studies had a negative result. Heaney [34] identified the most common flaw “baseline status” and the most pernicious flaw “(in)effective dosing”. However we found other flaws: not measuring 25OHD levels throughout the study limits the ability to know if the 25OHD level actually changed. In this case, there would be no reason to believe that the intervention caused a biological difference in Vitamin D levels between intervention and control groups. We also found more fundamental biological flaws where the intervention was not Vitamin D but calcium, and caused a decreased in the 25OHD level. These two studies were included in previous systematic reviews but rejected by this review.

The finding that meta-analyses for studies with biological flaws had the statistically significant effect of increasing depression, may lead to a conclusion that some of these trials led to levels for Vitamin D above the therapeutic range. This would be supported by a recent paper indicating that the therapeutic range for 25OHD in depression is 50 and 85 nmol/L [55].

It may be argued that meta-analysis including flawed RCTs reflect the trial methodology more than the efficacy of the intervention, leaving reviewers unable to make valid conclusions about efficacy [34], resulting in uncertainty amongst researchers and clinicians. This has led to calls for more RCTs and less “torturing of the data” by meta-analysis [56]. However, as this review demonstrates, it is excluding biological flaws that will lead to greater understanding of Vitamin D, not simply increasing the quantity of studies.

We note that biological flaws are more frequent in recent studies; this may be due to the belief that vitamins exert a function beyond deficiency. Hence RCTs should test whether using supplementation to correct deficiency is beneficial, rather than testing whether additional supplementation on top of the recommended doses is beneficial in reducing disease [57]. Thus, it is unremarkable that Vitamin D supplementation would not benefit a population that are not deficient or where the dose was ineffective. To test the hypothesis that correcting Vitamin D deficiency leads to an improvement in depression, it is critical to exclude biological flaws from future studies.

The effect size for Vitamin D in depression demonstrated in this meta-analysis is comparable with the effect of anti-depressant medication, an accepted treatment for depression. Should these results be verified by future research, these findings may have important clinical and public health implications.
